# Influence of Psychological Factors in Breast and Lung Cancer Risk – A Systematic Review

**DOI:** 10.3389/fpsyg.2021.769394

**Published:** 2022-01-03

**Authors:** Maria Angelina Pereira, António Araújo, Mário Simões, Catarina Costa

**Affiliations:** ^1^Institute of Biomedical Sciences Abel Salazar, University of Porto, Porto, Portugal; ^2^Department of Medical Oncology, Centro Hospitalar Universitário do Porto, Porto, Portugal; ^3^Laboratory of Mind-Matter Interaction with Therapeutic Intention, Faculty of Medicine, University of Lisbon, Lisbon, Portugal; ^4^Clínica de Saúde Ser e Viver, Maia, Portugal

**Keywords:** psychological factors, adverse life events, trauma, grief, depression, breast cancer risk, lung cancer risk, oncology

## Abstract

**Introduction:** In 2020, according to the Global Cancer Observatory, nearly 10 million people died of cancer. Amongst all cancers, breast cancer had the highest number of new cases and lung cancer had the highest number of deaths. Even though the literatures suggest a possible connection between psychological factors and cancer risk, their association throughout studies remains inconclusive. The present systematic review studied the connection between psychological factors and the risk of breast and lung cancer, prior to a cancer diagnosis. The psychological factors of trauma, grief, and depression were studied.

**Methods:** The current systematic review was carried out across multiple databases in two phases, an initial exploratory research in June 2020, refined with a second electronic research in December 2020. The inclusion criteria included studies describing the association between trauma, posttraumatic stress disorder (PTSD), grief, and depression with breast and lung cancer risk. The psychological data collection must have been carried out prior to a confirmed breast or lung cancer diagnosis, and accessed through self-report measures, questionnaires, clinical interviews, or clinical diagnoses. Study reports had to contain information about the incidence of cancer and effect size. The exclusion criteria were studies in which psychological factors were collected after cancer diagnosis.

**Results and Conclusion:** A total of 26 studies were included. Although non-consensual, the findings from the present systematic review suggest that, in addition to the known risk factors, psychological factors may play an important role in the etiology of both breast and lung cancer. To include psychological factors as a variable that affects cancer development may be fundamental to opening new avenues for prevention and intervention.

**Systematic Review Registration:** [www.ClinicalTrials.gov], identifier [CRD42020209161].

## Introduction

Cancer is now the second leading cause of death in the world according to the World Health Organization [WHO] (2020) estimates. In 2020, according to the Global Cancer Observatory (GCO) (2020c) nearly 10 million people died of cancer. Amongst all cancers, both sexes and all ages, breast cancer had the highest number of new cases, nearly 2.3 million (11.7%) in an estimated 19.3 million worldwide, and was fifth in number of deaths (6.9%) Global Cancer Observatory (GCO) (2020a). Lung cancer was second in number of new cases (11.4%) and the first in number of deaths, almost 1.8 million (18%) Global Cancer Observatory (GCO) (2020b).

Europe, considering the cancer statistics worldwide by region, has the second highest number in incidence, mortality and 5-year prevalence in all cancers, including breast and lung cancers [Global Cancer Observatory (GCO), 2020a,b, c]. Although Europe has only 9.7% of the world population, the estimated percentage of new cases was 22.8% (Sung et al., 2021). In Portugal Global Cancer Observatory (GCO) (2020d), the total estimated number of new cancers cases was 60,467, with a total number of 30,168 deaths from all cancers. Breast cancer is second to colorectum cancer with a number of 7,041 (11.6%) new cases, and lung cancer is fourth with 5,415 (9%) new cases. Breast cancer was the most common in females, all ages. Lung cancer was the third most frequent in males and in females, all ages.

The major cancer risk factors considered by World Health Organization [WHO] (2021) and widely investigated are the use of tobacco (Kim et al., 2014, 2018; Drope et al., 2018), alcohol intake (Oyesanmi et al., 2010; Bagnardi et al., 2015; Sun et al., 2020), unhealthy diet (WCRF, 2018; Morze et al., 2021; Tran et al., 2021), and lack of physical activity (Boyne et al., 2018; Chan et al., 2019; McTiernan et al., 2019). The WHO also considers air pollution and chronic infections as risk factors. Despite findings suggesting the association between psychological factors and development of cancer, mental health problems have not been recognized as risk factors of cancer. Even though the WHO states that “there is no health without mental health” (World Health Organization [WHO], 2018), highlighting the association of mental and physical health.

Psychological and emotional distress from exposure to adverse childhood experiences (ACEs), adverse life events, or traumatic stressors can cause psychological trauma and associated posttraumatic stress disorder (PTSD), pathological grief, and depression (Kessler et al., 2010; Trickey et al., 2012; Wang et al., 2018). Risk factors for the severity of impact of adverse/traumatic events include: prior trauma (Breslau et al., 1999; Hughes et al., 2017); gender (Tolin and Foa, 2006; Gallo et al., 2018); age (Willis et al., 2018); individual vulnerability (Bomyea et al., 2012); heredity factors (Koenen et al., 2002; Bomyea et al., 2012; Wang et al., 2018); family or social functioning and support (Boscarino, 1995; Trickey et al., 2012; Stevens and Jovanovic, 2019); and, social variables such as ethnic, demographic, and socioeconomic factors (Kosidou et al., 2011; Santini et al., 2021), among others. One or more of these risk factors can lead to psychopathology and physical disease.

Psychopathology can be triggered by psychological trauma, grief, and depression, causing alterations in neuropsychological functioning, cognitive processing, emotional regulation, and in adjustment, among other processes (Kessler et al., 2010; Bomyea et al., 2012; Carr et al., 2013; McKay et al., 2021). Psychological theories and underlining psychodynamic processes associated to traumatic events include alterations in cognitive, emotional, identity, personality, and relational (socioemotional) development. These alterations cause vulnerability, emotional instability, difficulties in self-regulation, alterations in adjustment, and poor social relationships (Bomyea et al., 2012; Carr et al., 2013). The psychological and interlinked physical symptoms associated to trauma, grief, and depression include: dysregulation of emotions and feelings (sadness, hopelessness, fear, anger, mistrust, and worry); progressive increase in anxiety, stress; depression; cognitive impairment; alterations in appetite; disturbance of sleep; psychomotor apathy/agitation; behavioral alterations; somatic symptoms; and functional impairment (Carr et al., 2013).

Physical disease has already been linked to the psychological factors of trauma, grief, and depression, and associated with higher rates of autoimmune disease, metabolic syndrome, coronary heart disease, respiratory disease, and some cancers (Boscarino, 2004; Miller et al., 2011; Hughes et al., 2017; Lopes et al., 2020; Santini et al., 2021). This risk is associated to the physiological alterations in neural, neuroendocrine, immune, and cardiovascular systems (Boscarino, 2004; Bomyea et al., 2012). Physiological functioning has been evaluated in studies of neurodevelopment and brain structure, in genetic and epigenetic research, among others.

Evidence of alterations in brain structure, activity, and connectivity have been pinpointed in the neurobiological research associated to trauma in childhood. Neurobiological and neuro-imaging studies showed functional alterations and structural alterations with volume changes in the prefrontal cortex (PFC) and hippocampus (Cassiers et al., 2018). Studies also showed functional alterations in emotion processing in the anterior cingulate cortex, amygdala, and hippocampus, and alterations in executive functions in the PFC and basal ganglia (Fox et al., 2010; Miller et al., 2011; Luby et al., 2017; Cassiers et al., 2018). The cortico-amygdala neural circuitry regulates vigilance and responses to threatening stimuli, while the cortico-basal ganglia circuitry regulates reward processing. The neural reward system is important in many psychological and behavioral processes, such as learning, social bonding, addiction, among others. It is also involved in grief, linked to the psychological and physiological regulatory role played by attachment figures in this system (Kakarala et al., 2020). Another important neurobiological process associated to trauma and depression implicates the dysregulation of the hypothalamic–pituitary–adrenal (HPA) axis, resulting in increased levels of cortisol and pro-inflammatory cytokines (Nusslock and Miller, 2016; Cassiers et al., 2018; Wang et al., 2018; Majd et al., 2020). Neurobiological studies identified various alterations in mechanisms that play a key role in the regulation of inflammation, including the sensitization of immune cells that initiate and sustain inflammation, the alteration in the sensitivity of monocytes to inhibition by glucocorticoids, the declining suppression of cytokine production by cortisol, among other processes linking the nervous, endocrine, and immune systems, that may lead to a chronic pro-inflammatory state (Danese and McEwen, 2012; Nusslock and Miller, 2016). Inflammation, which plays an essential role as the pathogenic link influencing all the systems, is considered the “common soil” of the multifactorial diseases and is known to play an important role in cancer development (Mantovani et al., 2008; Miller et al., 2011; Scrivo et al., 2011; Danese and McEwen, 2012).

Genetic and epigenetic studies associated to trauma showed changes in specific genes that regulate neural circuits. Some of these genes regulate function in the serotonin, catecholamines, and glucocorticoids systems, which are also implicated in depression (Bomyea et al., 2012; Majd et al., 2020). Genetic research focusing on epigenetic factors has, among other investigation, linked changes in DNA methylation (DNAm) (hypermethylation and hypomethylation) to pathways for development of disease in the different systems (Bomyea et al., 2012; Ridout et al., 2018; Lang et al., 2020; Czamara et al., 2021; Parade et al., 2021). Czamara et al. (2021) demonstrated that the effects of the interaction of genotype and exposure to childhood adversity had the highest influence on DNAm variability. The systematic review by Lang et al. (2020) explored studies with other markers of biological changes to ACEs, namely inflammatory markers such as tumor necrosis factor alpha (TNF-α) and interleukin-6 (IL-6), epigenetic modification by methylation [methylation of glucocorticoid receptor (GR) gene – *NR3C1*, methylation of the brain-derived neurotrophic factor (BDNF), methylation of the serotonin signaling genes], microRNA 124-3, interferon gamma (IFN-γ), corticotrophin releasing hormone (CRH), telomere length, and structural changes using functional MRI brain imaging. The inflammatory cytokines TNF-α and IL-6, were also identified in depression and are key endogenous factors in cancer-related inflammation (Mantovani et al., 2008; Majd et al., 2020).

The association between psychological factors, such as depression, anxiety, stress or personality, and cancer has been studied for a long time with controversial results (Nakaya et al., 2010; Lemogne et al., 2013a; Lin et al., 2013; Jia et al., 2017; Afrisham et al., 2019; Bahri et al., 2019; Abate et al., 2020). The psychological factors of trauma and grief have been less studied.

In the association of adverse life events/trauma, Bahri et al. (2019) in the systematic review and meta-analysis of cohort studies published in 2019 found that stressful life events “slightly increase the risk” of breast cancer with a pooled risk ratio (RR) of 1.11 [95% confidence interval (CI) = 1.03–1.19]. Lin et al. (2013) found a 1.5-fold greater risk for “striking life events” with odds ratio (OR) of 1.51 (95% CI = 1.15–1.97) and a 2.0-fold greater risk for “severe striking life events” with OR of 2.07 (1.06–4.03). The meta-analysis by Duijts et al. (2003) showed no association between adverse life events and breast cancer, except for the event of the death of a spouse with a moderate risk (OR = 1.77, 95% CI = 1.31–2.40) for breast cancer.

No published systematic reviews or meta-analysis on the association of grief and cancer risk were found.

In relation to the association of depression and cancer risk, the meta-analysis by McGee et al. (1994) concluded with “a small, but marginally statistically significant association between depression and the later development of cancer”. Oerlemans et al. (2007) confirmed this result on overall cancer with a summary RR of 1.19 (95% CI = 1.06–1.32) and found, in the analysis of studies with a follow-up of 10 or more years, that the risk between depression and subsequent breast cancer increased to a significant RR of 2.50 (1.06–5.91). On the other hand, Sun et al. (2015) found no significant association between depression and breast cancer. In more recent systematic reviews, Jia et al. (2017), found a significant association with overall cancer risk (RR = 1.15, 95% CI = 1.09–1.22), with liver cancer (RR = 1.20, 95% CI = 1.01–1.43), and lung cancer (RR = 1.33, 95% CI = 1.04–1.72). Wang et al. (2020) found, associated to clinically diagnosed depression a significantly increased risk of cancer incidence (adjusted RR = 1.13, 95% CI = 1.06–1.19). In terms of specific cancer sites, significant associations to lung cancer were observed (RR = 1.41, 95% CI = 1.17–1.69).

Results of the studies are inconsistent, and one explanation is that the epidemiological data comes from retrospective case-control studies open to recall bias (McGee et al., 1994). Important explanations included heterogeneity between studies due to demographics, study design, population studied, and assessment measures among other limitations (Wang et al., 2020).

The present systematic review aimed to study the connection between psychological factors and the risk of breast and lung cancer. The psychological factors of trauma, grief, and depression were studied. These factors may be present without a formal clinical diagnosis, and cause suffering, functional limitations, and morbidity. To study the association between psychological factors and the risk of breast and lung cancer, six research questions were defined as follows: is there a consensual association between (1) psychological trauma and breast cancer risk? (2) grief and breast cancer risk? (3) depression and breast cancer risk? (4) psychological trauma and lung cancer risk? (5) grief and lung cancer risk? and (6) depression and lung cancer risk?

Only studies in which the study variables were obtained before diagnosis of breast or lung cancer were included.

## Methods

The systematic review protocol was registered with the International prospective register of systematic reviews (PROSPERO) and published without review on October 16, 2020 with registration number CRD42020209161.

The current systematic review was conducted using PRISMA statement guidelines for systematic reviews (Moher et al., 2009) and followed the recommendations of the Cochrane Collaboration for systematic reviews (Higgins et al., 2020).

### Search Strategy

The current systematic review was carried out in two phases. The initial exploratory research was conducted at PubMed/MEDLINE database, from June 1, 2020 to July 28, 2020, using the following descriptors: psychological factor, mental health, negative life events, trauma, PTSD, grief, depression, breast cancer risk, and lung cancer risk. The search was refined by combinations of these descriptors and the Boolean operator (AND). The MeSH terms: trauma, grief, and mental health were also used. No temporal or language restrictions were used.

The second electronic research was performed in PubMed/MEDLINE, Scopus, Web of Science, Scielo, and APA PsyInfo databases, from December 1, 2020, to December 31, 2020, using the following descriptors: adverse life events, psychological trauma, post-traumatic stress disorder, grief, bereavement, mourning, depression, depressive disorder, depressive symptoms, breast cancer risk, and lung cancer risk. The search was refined by the combinations of those descriptors and two Boolean operators (AND) and (OR). No restrictions were made on dates or language.

The identified studies were reviewed independently by two reviewers who independently screened all titles, abstracts, and full-text articles. Any disagreement was resolved by consensus with the other authors. Reasons for excluding studies were documented.

### Eligibility Criteria

The inclusion criteria included all relevant studies describing the association between trauma, PTSD, grief, and depression with breast and lung cancer risk. It included published studies with participants with suspected breast or lung cancer. The psychological data collection must have been carried out prior to a confirmed breast or lung cancer diagnosis and accessed through self-report measures, questionnaires, clinical interviews, or clinical diagnoses. Study reports had to contain information about the incidence of cancer and effect size. The exclusion criteria were studies in which psychological factors were collected after cancer diagnosis.

### Eligible Studies

**FIGURE 1:**
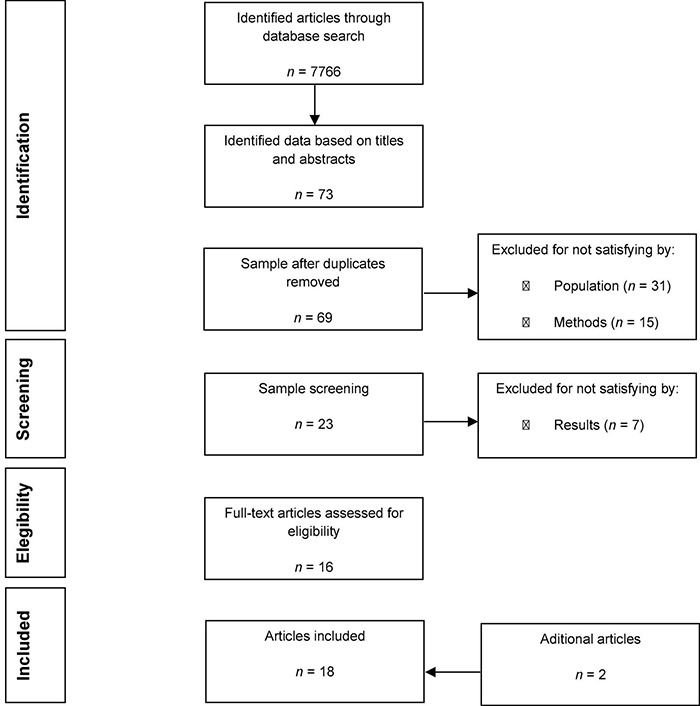
Flow diagram of the initial exploratory research selection.

A total of 73 articles were identified from PubMed/MEDLINE database, after the first round of screening based on titles and abstracts with inclusion criteria. After examining the articles in more detail, 57 articles were excluded – four per repetition, six for not reporting cancer and effect size, and the rest (47 articles) for not accessing the psychological variables under study before a breast or lung cancer diagnosis. Only 16 articles met the inclusion criteria. Later, another two articles were identified by searching the reference lists. In total, 18 articles were included in this systematic review (Figure 1).

**FIGURE 2:**
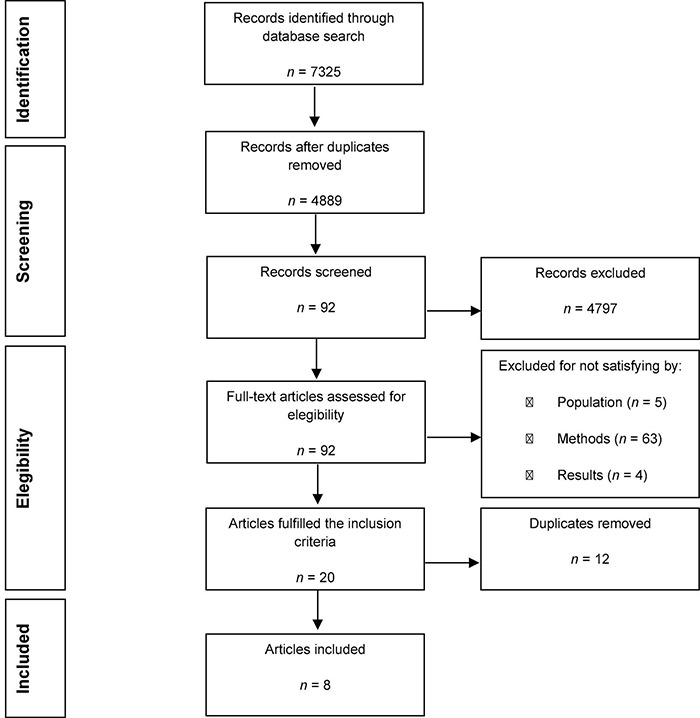
Flow diagram of the second study selection.

In the second phase of the review, 7,325 articles were identified (232 APA PsyInfo; 1,943 PubMed/MEDLINE; 1 Scielo; 501 Scorpus; and 4,648 Web of Science). A total of 2,436 duplicates were removed using EndNote 20 and the titles and abstracts of articles were then evaluated. In total, 92 articles were found to be relevant. Full-length papers of the shortlisted articles were assessed for the eligibility criteria and 20 articles fulfilled the inclusion criteria. Subsequently, both searches were joined, and 12 duplicates were removed (Figure 2).

A final total of 26 articles were considered for the systematic review.

### Data Extraction and Quality Assessment

A standardized table was used to extract the relevant information from all the included studies (Table 1): author(s); country; population; psychological factors under study; type(s) of cancer; measures; follow-up years; adjusted covariates; quality; cancer; and effect size. Adjusted RRs were used to measure effect size with 95% CI for cancer risk incidence. The quality of included studies was assessed using the Newcastle Ottawa Scale (NOS) (Wells et al., 2020) as recommended by the Cochrane Non-Randomized Studies Methods Working Group for non-randomized studies, including case-control and cohort studies. The scale accesses three criteria: (1) quality and representativeness of sample (4 items, each with a possible score of one point); (2) comparability between groups (1 item, with a possible score of two points); and (3) verification of the exposure or result of interest (3 items, each with a possible score of one point). The total score of the scale ranges from 0 to 9. Higher scores indicate higher quality. The assessment was done by two researchers.

**TABLE 1:** Characteristics of included studies – psychological factors and cancer risk.

Author(s)	Country	Population	Psychological Factors	Type(s) of Cancer	Measures	Follow-up years	Adjusted Covariates	Quality	Cancer and Effect Size (95% CI)
Trudel-Fitzgerald et al. (2020)	United States	121 700 females	Depressive symptoms	Lung	MHI-5.	24	Age; husband’s education; education; marital status; diet quality; physical activity; shift work; smoke; and exposure to smoke.	9	Females with severe depressive symptoms had higher lung cancer risk (RR = 1.25, 95% CI = 1.04–1.51, *p* < 0.05). No significant associations were observed between moderate depressive symptoms and lung cancer risk (RR = 0.99, 95% CI = 0.84–1.16, *p* = 0.01).
Kaster et al. (2019)	Canada	36 309 participants (20 310 females)	Trauma PTSD	Breast Others	Clinical interview; DSM-V.	1	Age; sex; ethnicity; marital status; education; household; drugs; alcohol; smoke; mood disorder; and anxiety disorder.	8	No significant associations were observed between trauma or PTSD and breast cancer risk in females (RR = 0.80, 95% CI = 0.55–1.17, *p* > 0.05; RR = 1.00, 95% CI = 0.49–2.04,*p* > 0.05), respectively.
Brown et al. (2016)	United States	71 439 females	Depressive symptoms	Breast	Burnam eight-item scale.	5	Age; BMI; alcohol; smoke; physical activity; parity; age at first birth; menopause; and race.	7	No association between depressive symptoms and the risk of total breast cancer (RR = 0.96, 95% CI = 0.85–1.08, *p* > 0.05), invasive breast cancer (RR = 0.98, 95% CI = 0.86–1.12, *p* > 0.05), or in situ breast cancer (RR=0.86, 95% CI = 0.65–1.14, *p* > 0.05) were found.
Reeves et al. (2018)	United States	238.129 females aged	Major Depression	Breast	MHI-5.	10	Age; use of antidepressants; history of breast in first degree relative; age at menarche; age at menopause; history of benign breast disease; hormone therapy; alcohol intake; and smoke.	6	No significant associations were observed between depression and breast cancer risk (RR = 1.13, 95% CI = 0.85–1.49, *p* > 0.05).
Mitchell et al. (2017)	United States	1945 females	Major Depression Dysthymia Other	Breast	DIS DSM-III Baseline questionnaire.	24	Age; race/ethnicity; socioeconomic status; and smoke.	7	No significant associations were observed between major depression (RR = 1.36, 95% CI = 0.31–5.94, *p* > 0.05) or dysthymia (RR = 1.14, 95% CI = 0.15–8.78, *p* > 0.05) and breast cancer risk.
Schoemaker et al. (2016)	United Kingdom	113.000 females	Adverse Life Events	Breast	Stress baseline questionnaire.	5	Age; premenopausal; absence of menstrual periods; death of mother or father with cancer; other family cancer history.	8	Overall breast cancer risk has no association with overall experienced frequency of stress (RR = 0.92, 95% CI = 0.83–1.03, *p* = 0.15) or continuously of stress (RR = 0.91, 95% CI = 0.73–1.15). An increased risk is observed with the death of a close relative (RR = 0.87, 95% CI = 0.78–0.97, *p* < 0.05).
Chang et al. (2015)	Korean	1,220,697 participants (797,959 males)	Depression	Breast Others	9-item depression questionnaire; DSM-IV; MIC data.	2	Sex; smoke; alcohol intake; exercise; body mass index; cholesterol; blood sugar; hypertension; and history of breast cancer family.	8	No significant associations were observed between overall depression and breast cancer risk (RR = 0.92, 95% CI = 0.82–1.02, *p* > 0.05).
Gradus et al. (2015)	United States	4131 participants	PTSD	Lung Breast Others	ICD-10.	16	Age; sex; and substance abuse.	8	An overall null association was found for PTSD and cancer (lung, RR = 1.3, 95%, CI = 0.73–2.0, *p* > 0.05; breast, RR = 1.2, 95% CI = 0.82–1.7, *p* > 0.05).
Hung et al. (2014)	Taiwan	20.033 participants (9702 male)	Depression Other	Lung Breast Others	Clinical diagnosis.	13	Age; sex; alcohol intake; and smoke.	8	Participants with depression had an increased risk for lung cancer (RR = 1.65, 95% CI = 1.25–2.17, *p* < 0.001) and breast cancer (RR = 2.25, 95% CI = 2.01–3.24, *p* < 0.001).
Lemogne et al. (2013a)	France	44.922 participants (31.411 males)	Depression Depression Mood	Breast Others	DSM-IV; clinical records; CES-D.	15	Age; sex; occupational grade; alcohol intake; fruit and vegetables consumption; smoke; weight; physical activity; and health status.	8	No compelling evidence for an association between depression or depression mood and breast cancer incidence (RR = 1.01, 95% CI = 0.66–1.55, *p* > 0.05).
Chen and Lin (2011)	Taiwan	778 participants	Depression	Breast Others	ICD-9CM.	5	Age; and sex.	8	Depression doesn’t show an increased risk for breast cancer in females (RR = 1.25, 95% CI = 0.42–3.76, *p* > 0.05).
Gross et al. (2010)	United States	3481 participants (1945 female)	Depression	Breast Lung Others	DIS; clinical interview; DSM-III.	24	Age; marital status; socioeconomic status; gender; smoke; and ethnicity.	8	Depression doesn’t show an increased risk for breast cancer in females (RR = 3.38, 95% CI = 0.83–13.76, *p* > 0.05) or for lung cancer risk e both sexes (RR = 0.82, 95% CI = 0.25–2.64, *p* > 0.05).
Goldacre et al. (2007)	United Kingdom	577.545 participants (19.153 males)	Depression Others	Lung Breast Others	Clinical interview – ICD 10.	36	Age; sex; and other pathologies identified.	9	Lung cancer was more common in those with depression (RR = 1.36, 95% CI = 1.12–1.48, *p* < 0.05). Breast cancer risk was not increased in participants with depression (RR = 0.95, 95% CI = 0.83–1.09, *p* > 0.05).
Aro et al. (2005)	Finland	10.892 females	Depression Negative Life Events Death loss	Breast	BDI; Modify coping with loss scale; cynical distrust 10-item scale.	9	Age; breast cancer family history; smoke; alcohol intake; physical exercise; parity; education; socioeconomic status; and controlling for area.	8	The psychological factors did not significantly predict breast cancer (lifetime serious illness: RR = 1.30, 95% CI = 1.04–1.84, *p* > 0.05; withdrawal coping: RR = 1.05, 95% CI = 0.76–1.46, *p* > 0.05; cynical distrust: RR = 1.23, 95% CI = 0.77–1.64, *p* > 0.05; emotion-focused coping: RR = 0.85, 95% CI = 0.60–1.19, *p* > 0.05; poor perceived health: RR = 0.75, 95% CI = 0.46–1.22, *p* > 0.05); and depression: RR = 1.8, 95% CI = 0.95–1.25, *p* > 0.05).
Ollonen et al. (2005)	Finland	115 females	Depression Others	Breast	Clinical interview; MADRS; BDI; FI.	2	Age; smoke; alcohol intake; family history of breast cancer; body weight; postmenopausal; and oral contraceptives.	7	Breast cancer increased slightly in participants with depression (RR = 1.1, 95% CI = 0.77–1.56, *p* = 0.51.
Montazeri et al. (2004)	Iran	3000 females aged [>18]	Depression Depression symptoms	Breast	Clinical interview; DSM-IV.	3	Age; age ate first menarche; age at first-time full-term pregnancy; family history of breast cancer; menopausal status; oral contraceptives use; and history of psychiatric medications.	8	Breast cancer risk increased when associated with a previous depression mood (RR = 1.90, 95% CI = 1.12–3.25, *p* < 0.001) and hopelessness (RR = 1.63, 95% CI = 1.05–2.56, *p* < 0.05).
Lillberg et al. (2003)	Finland	10.808 females	Adverse Life Events	Breast	Holmes and Rahe scale; SWLS.	5	Age; body mass index; alcohol intake; smoke; use of oral contraceptives; physical activity; and breast family history.	7	Breast cancer risk increased in those who reported overall stressful life events (RR = 1.07, 95% CI = 1.00–1.15, *p* < 0.50). This risk rises when major life events were considered (RR = 1.35, 95% CI = 1.09–1.67. *p* < 0.001). Particularly, divorce/separation (RR = 2.28, 95% CI = 1.25–4.07, *p* < 0.05), death of a husband (RR = 2.00, 95% CI = 1.03–3.88, *p* < 0.05), and death of a close friend (RR = 1.36, 95% CI = 1.00–1.88, *p* < 0.05).
Nyklícek et al. (2003)	Netherlands	5191 females	Depressive symptoms	Breast	EDS.	2	Age; age at menarche; breast cancer history; menopausal age; body mass index; education; history of breastfeeding; oestrogens use; physical exercise; alcohol intake; and other pathologies.	7	Participants with depressive symptoms had a lower risk of breast cancer (RR = 0.29, 95% CI = 0.09–0.92, *p* = 0.04).
Dalton et al. (2002)	Denmark	89.491 participants (57.320 males)	Depression Other	Lung Breast Others	ICD-8; clinical interview.	25	Sex; alcohol intake; smoke; and number of hospital admissions for mental health.	8	Depression doesn’t show an increased risk for lung cancer in both sexes (RR = 1.59, 95% CI = 1.17–1.72, *p* > 0.05) and breast cancer in females (RR = 1.06, 95% CI = 0.98–1.76, *p* > 0.05).
Gallo et al. (2000)	United States	3109 participants (1202 male)	Major depression Dysphoric episode	Lung Breast Others	QIDS-SR.	13	Age; sex; alcohol intake; smoke; ethnicity; marital status; and education.	8	Among women with major depression, the risk of breast cancer was increased (RR = 3.8, 95% CI 1.0–14.2, *p* < 0.001), but not for dysphoria episode (RR = 1.4, 95% CI = 0.5–3.4, *p* > 0.05). No overall association of major depression (RR = 1.0, 95% CI = 0.1–7.7, *p* > 0.05) or dysphoric episode (RR = 0.4, 95% CI = 0.1–1.5, *p* > 0.05) with lung cancer risk incidence.
Jacobs and Bovasso (2000)	United States	1213 females	Depression Stressful Life Events	Breast	DIS; clinical interview.	2	Age; drug abuse; smoke; heart disease; diabetes; stroke and hypertension; other disease family history.	8	Maternal death in childhood predicted risk of breast cancer (RR = 2.56, 95% CI = 1.59–4.35, *p* < 0.001) and chronic depression with severe episodes (RR = 14.0, 95% CI = 1.59–4.35, *p* < 0.05).
Hjerl et al. (1999)	Denmark	66.648 females	Dysthymia Other	Breast	ICD-8	25	Age; place of residence; alcohol; and smoke.	8	No increased breast cancer risk was observed in participants with dysthymia (RR = 1.10, 95% CI = 0.95–1.25, *p* > 0.05).
Pennix et al. (1998)	United States	4825 participants (1708 male)	Depression Mood	Lung Breast Others	CES-D; admissions survey.	6	Age; sex; smoke; alcohol intake; physical disability.	8	Lung cancer increased in participants chronically depressed (RR = 2.10, 95% CI = 0.49–8.92, *p* < 0.001). No increased breast cancer risk was observed (no depressive cases).
Knekt et al. (1996)	Finland	7018 males	Depression	Lung	Clinical interview; 36-item version of GHQ; short version of PSE.	14	Age; sex; education; smoke; alcohol intake; body mass index; serum cholesterol; leisure-time exercise; and general health.	7	Depressiveness was associated with the incidence of lung cancer (RR = 3.38, 95% CI = 1.09–15.7, *p* < 0.001). Clinical depression has no association with lung cancer risk (RR = 1.65, 95% CI = 0.60–4.58, *p* > 0.05).
Jasmin et al. (1990)	France	2298 females	Depressive symptoms Grief Others	Breast	Clinical interview.	4	Age; family history of breast cancer; oral contraceptives use; premenopausal status; and other pathologies.	9	No significant associations were observed between depression and breast cancer risk (latent depression: RR = 1.6, 95% CI = 0.6–4.5, *p* > 0.05; essential depression: RR = 1.2, 95% CI = 0.4–3.5, *p* > 0.05; acute depression: RR = 1.3, 95% CI = 0.4–3.8, *p* > 0.05). The unsolved recent grief was found to be related to increase breast cancer risk (RR = 8.2, 95%, CI = 1.0–73.5, *p* = 0.05).
Hahn and Petiti (1988)	United States	8932 females	Depression	Breast	MMPI.	4	Age; use of contraceptives and other sex steroid hormones; smoke; alcohol intake; and other unspecified medical history.	7	No increased breast cancer risk was observed in participants with depression (RR = 1.4, 95% CI = 0.8–2.4, *p* > 0.05).

*Note: When hazard ratios and incidence risk ratios were reported, we considered them directly as risk ratios (RRs).*

## Results

**FIGURE 3:**
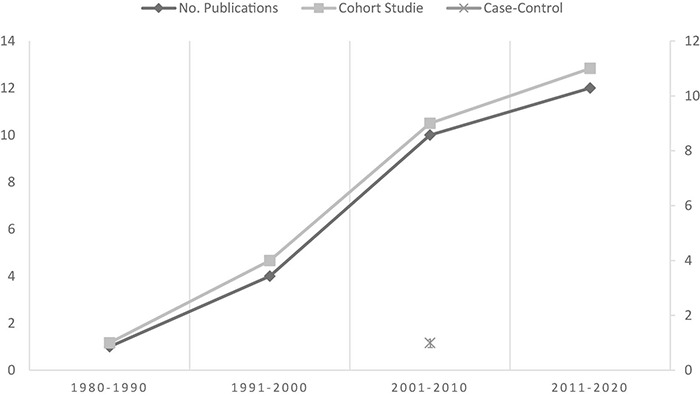
Distribution of the included studies by date and type of research.

The systematic review recognized 26 studies, 25 cohort and one case-control, that investigated the incidence of breast and lung cancer in people with history of adverse life events or trauma, grief, and depression at baseline. These 26 studies included a total of 2,554,762 participants (943,056 males), range 115 (Ollonen et al., 2005) to 1,220,697 (Chang et al., 2015). They were conducted between 1988 and 2020 (Figure 3) in Canada (*n* = 1), Denmark (*n* = 2), Finland (*n* = 4), France (*n* = 2), Iran (*n* = 1), South Korea (*n* = 1), Netherlands (*n* = 1), Taiwan (*n* = 2), United Kingdom (*n* = 2), and United States (*n* = 10). The follow-up time of these studies ranged from 1 (Kaster et al., 2019) to 36 years (Goldacre et al., 2007). Studies reported the cancer effect size of breast (*n* = 17), lung (*n* = 2), or both cancers (*n* = 7), after adjusted covariates.

The psychological factors were accessed through clinical interviews, clinical diagnoses based on ICD or DSM and self-report measures, namely the Beck Depression Inventory (BDI), Burnam eight-item scale, Centre of Epidemiologic Studies-Scale (CES-D), Cynical Distrust 10-item Scale, Diagnostic Interview Survey (DIS), Edinburgh Depression Scale (EDS), 36-item version of General Health Questionnaire (GHQ), Holmes and Rahe Scale, Life Events Scale in the Epidemiological Risk Factor (ERF), Mental Health Inventory-5 (MHI-5), Minnesota Multiphasic Inventory (MMPI), Modify Coping with Loss Scale, Modify Haan Coping and Stress Inventory (FI), Montegomery-Asberg Depression Rating Scale (MADRS), short-form of the Present State Examination (PSE), and Quick Inventory of Depression Symptomatology (QIDS-SR) (Table 1).

Overall, 12,962/2,554,762 cases of trauma, 1,667/2,554,762 cases of grief, and 694,537/2,554,762 cases of depression were reported at baseline (Table 2).

**TABLE 2:** Summary of results.

Results	
**Included studies**	

Total	*N* = 26
Years	1988–2020
Follow-up years	1–36

**Participants**	

Total	2,554,762 participants
Males	943,056 males

**Location of studies**	

Canada	*n* = 1
Denmark	*n* = 2
Finland	*n* = 4
France	*n* = 2
Iran	*n* = 1
Korea	*n* = 1
Netherlands	*n* = 1
Taiwan	*n* = 2
United Kingdom	*n* = 2
United States	*n* = 10

**Cancer effect size**	

Breast cancer	*n* = 17
Lung cancer	*n* = 2
Both cancers	*n* = 7

**Psychological factors**	

Trauma	*n* = 12,962 at baseline
Grief	*n* = 1,667 at baseline
Depression	*n* = 694,537 at baseline

*N, sample; n, number of elements.*

Regarding breast cancer, five articles studied the association with trauma, two with grief, and 20 with depression. Considering lung cancer, one article investigated the association with psychological trauma, and eight investigated the association with depression. No study included in this systematic review investigated the association between grief and lung cancer. The positive statistically significant results found are presented in relation to the six research questions defined.

### Association Between Psychological Trauma and Breast Cancer Risk

Five studies explored the association between psychological trauma and breast cancer risk. Of these studies, three reported a positive statistically association among female participants: Jacobs and Bovasso (2000); Lillberg et al. (2003), and Schoemaker et al. (2016). The most significant adverse life events/trauma reported relate to: death of a close relative (RR = 0.87, 95% CI = 0.78–0.97, *p* < 0.05) (Schoemaker et al., 2016); divorce/separation (RR = 2.28, 95% CI = 1.25–4.07, *p* < 0.05), death of a spouse (RR = 2.00, 95% CI = 1.03–3.88, *p* < 0.05), and death of a close friend (RR = 1.36, 95% CI = 1.00–1.88, *p* < 0.05) (Lillberg et al., 2003). Maternal death in childhood was also reported (RR = 2.56, 95% CI = 1.59–4.35, *p* < 0.001) (Jacobs and Bovasso, 2000).

### Association Between Grief and Breast Cancer Risk

Two studies investigated the association between grief and breast cancer risk. Only one study, Jasmin et al. (1990) reported a positive statistically association particularly with unsolved recent grief among females (RR = 8.2, 95% CI = 1.0–73.5, *p* < 0.05).

### Association Between Depression and Breast Cancer Risk

Twenty studies examined the association between depression and breast cancer risk. Only five studies reported a statistically significant association, among females: Gallo et al. (2000) (RR = 3.8, 95% CI = 1.0–229 14.2, *p* < 0.001); Hung et al. (2014) (RR = 2.25, 95% CI = 2.01–3.24, *p* < 0.001); Jacobs and Bovasso (2000) (RR = 14.0, 95% CI = 1.59–4.35, *p* < 0.05); Montazeri et al. (2004) (RR = 1.90, 95% CI = 1.12–3.25, *p* < 0.001); and Ollonen et al. (2005) (RR = 1.1 95% CI = 0.77–1.56, *p* < 0.05). The depression measured in these five studies was clinically defined, except for Montazeri et al. (2004) who differentiated depressive symptoms (depressive mood (RR = 1.90, 231, 95% CI = 1.12–3.25, *p* < 0.001) and hopelessness (RR = 1.63, 95% CI = 1.05–2.56, *p* < 0.51).

### Association Between Psychological Trauma and Lung Cancer Risk

One study, Gradus et al. (2015), explored the association between psychological trauma and lung cancer. However, no positive statistically significant associations were reported in both sexes.

### Association Between Grief and Lung Cancer Risk

Considering the association between grief and lung cancer, no studies that met the inclusion criteria were found.

### Association Between Depression and Lung Cancer Risk

Eight studies examined the association between depression and lung cancer. Five studies provided evidence for a positive statistically significant associations in both sexes: Goldacre et al. (2007) (RR = 1.35, 95% CI = 1.12–1.48, *p* < 0.05); Hung et al. (2014) (RR = 1.65, 95% CI = 1.25–2.17, *p* < 0.001); Knekt et al. (1996) (RR = 3.38, 95% CI = 1.09–15.7, *p* < 0.001); Pennix et al. (1998) (RR = 2.10, 95% CI = 0.49–8.92, *p* < 0.001); and Trudel-Fitzgerald et al. (2020) (RR = 1.25, 95% CI = 1.04–1.51, *p* < 0.01). The depression measured was clinically defined (Table 3).

**TABLE 3:** Summary of included studies with statistically significant associations.

Psychological factors	Breast cancer studies	Lung cancer studies
Trauma	Jacobs and Bovasso, 2000; Lillberg et al., 2003; Schoemaker et al., 2016	
Grief	Jasmin et al., 1990	
Depression	Gallo et al., 2000; Jacobs and Bovasso, 2000; Montazeri et al., 2004; Ollonen et al., 2005; Hung et al., 2014	Knekt et al., 1996; Pennix et al., 1998; Goldacre et al., 2007; Hung et al., 2014; Trudel-Fitzgerald et al., 2020

*All studies presented show statistically significant associations, p < 0.05. Twenty-six studies were analyzed.*

## Discussion

The main goal of this systematic review was to identify, select, and evaluate studies that investigated the association between psychological factors and the risk of breast and lung cancer in people without a diagnosis of cancer. In this review 26 studies were analyzed, 25 cohort and one case-control. The epidemiological evidence identified was diversified by country of study, cancer subtype, time of follow-up, psychological factors at baseline, measures used, and control for confounding factors. The studies included individuals with different age groups, sex, and sociodemographic characteristics. The psychological data collection was carried out in most studies through self-reporting measures scientifically validated for the populations studied, while in other studies data was collected through clinical interviews using the diagnostic criteria of ICD and DSM. All studies adjusted their statistical models to the main variables known as risk factors, for both cancer subtypes (World Health Organization [WHO], 2020). Six research questions were defined.

The first research question sought to understand whether there is a consensual association between psychological trauma and breast cancer risk. This review shows evidence that some adverse life events/trauma are statistically associated to an increased risk of breast cancer in females and shows that death loss during the different phases of development has a well-established long-term impact on physical health (Schoemaker et al., 2016). In particular, the self-reported events perceived at baseline with psychological distress stand out, such as the death of a friend, close family member, or spouse (Jacobs and Bovasso, 2000; Lillberg et al., 2003; Schoemaker et al., 2016). These results corroborate the associations already found in previous systematic reviews (Duijts et al., 2003; Lin et al., 2013). Death as an adverse life event/trauma seems to contribute the most to breast tumor development (Bahri et al., 2019). Therefore, psychological treatment of adverse life events/trauma can reduce the occurrence of breast cancer in females. Bahri et al. (2019) already warned of the need to intervene psychologically after the occurrence of an adverse life event/trauma. Experiencing the death of a significant person seems to be enough to pose risks for health problems. Considering the neurobiology, alterations that occur with adverse life events/trauma cause dysregulation of the HPA axis, resulting in increased levels of cortisol and inflammatory cytokines, which are key factors in cancer-related inflammation (Mantovani et al., 2008; Scrivo et al., 2011). Even though a solid causal association to inflammation has not yet been specified, inflammatory mediators seen in chronic inflammatory responses are present in breast cancer (Mantovani et al., 2008). In fact, it is established that non-steroidal anti-inflammatory drugs reduce the risk of developing breast cancer and reduce the mortality caused by these cancers (Mantovani et al., 2008). Psychological trauma causes neurodevelopmental changes and consequently alterations in neuropsychological functioning, which influences and is also influenced by the nervous system and the interlinked endocrine and immune systems that are also involved in cancer risk (Carr et al., 2013; McKay et al., 2021).

The aim of the second question was to explore the association between grief and breast cancer risk. This association has apparently been poorly studied empirically. In the present review, we found only two studies which met inclusion criteria. Only one described a positive significant statistical association, in relation to unsolved recent grief among females, which makes it impossible to generalize results (Jasmin et al., 1990). Death loss appears to play a role in the risk of breast cancer, when analyzed as an adverse life event (Schoemaker et al., 2016), as reflected in the first research question. Therefore, it would be expected to find statistically significant positive associations in both studies analyzed, which did not occur. The reasons for these disparities are unclear, but methodological differences in defining grief may be the contributing factor. Thus, it would be interesting that further empirical studies explore the association between grief and the subsequent development of breast cancer to differentiate between grief and death loss as an adverse event.

The third question reviewed the existence of an association between depression and breast cancer risk. Our results show that depression was the most studied psychological factor, being associated with a small to moderate breast cancer risk in females (Jacobs and Bovasso, 2000; Montazeri et al., 2004; Ollonen et al., 2005; Hung et al., 2014; Chang et al., 2015). Thus, these results support the evidence already described in the literature, that depression is a factor to be considered in the subsequent development of overall cancer (McGee et al., 1994; Oerlemans et al., 2007). However, 15 of the 20 studies included in this systematic review do not support this relationship and meet the results found in previous reviews that point to non-consensual results, especially in breast cancer (Oerlemans et al., 2007; Sun et al., 2015; Jia et al., 2017). Several hypotheses can be put forward to explain these results, namely in relation to the measures used by each study to access depression. Time of follow-up may be a significant factor. Oerlemans et al. (2007) found that in studies with a follow-up of 10 or more years, the risk between depression and subsequent breast cancer increased to a significant statistical association. Likewise, other variables that were not evaluated in all of the studies may be influencing these results, such as duration of depression, use of psychiatric medication, among others. The duration of depression and the use of psychiatric medication influence the neurobiology and physiology, involving the nervous (HPA axis), endocrine, and immune systems, and may alter the pro-inflammatory state, all of which play an important role in cancer development (Mantovani et al., 2008; Scrivo et al., 2011).

The fourth question sought to understand whether there is an association between adverse life events or psychological trauma, and risk of lung cancer. No statistically significant association between adverse life events/trauma and lung cancer risk was reported (Gradus et al., 2015). However, these results cannot be generalized due to lack of studies.

The fifth questioned the possible association between grief and lung cancer risk. However, no studies that met the inclusion criteria were found with reference to this association.

Lastly, the sixth research question sought to understand whether there is an association between depression and the risk of lung cancer. Five of the eight studies report a significant statistical association regarding depression, among both sexes (Knekt et al., 1996; Pennix et al., 1998; Goldacre et al., 2007; Chang et al., 2015; Trudel-Fitzgerald et al., 2020). These results are in line with what was previously described in preceding systematic reviews (Jia et al., 2017; Wang et al., 2020). Large sample studies are required to further research and clarify these associations. However, the negative associations found also need to be reflected on. The association of depression to the use of tobacco has been studied. The use of tobacco is also a known risk factor in lung cancer etiology [Drope et al., 2018; World Health Organization [WHO] (2021)]. It is important to clarify whether the association depression-lung cancer is being mediated by the use of tobacco. This question has already been raised by Trudel-Fitzgerald et al. (2020).

### Clinical Implications

Breast cancer among females, associated to depression at baseline is one of the most extensively studied cancer subtypes, followed by the study of the association with negative life events or trauma. This shows the interest of the scientific community in understanding the link between psychological factors and breast cancer. On the other hand, studies associated to grief seem to have very little expression. Which is a paradox, considering that loss and grief are a part of the reality of each individual. The presence of psychological symptoms influences treatment response and prognosis of cancer.

Research of the association of psychological factors with lung cancer is still at an early stage. Nonetheless, many publications reporting the prevalence of depression at the baseline were found, which shows that psychological distress is very present in these populations even prior to a diagnosis of cancer. This psychological suffering may be aggravated when a cancer diagnosis occurs and influence even more the prognosis and mortality (Wang et al., 2020).

Our findings point out that early detection of the mental status and appropriate intervention can influence treatment and prognosis of cancer and thereby save resources for health systems in the treatment of cancer. Furthermore, they pave the way for new psychological procedures that consider the mind-body connection. Associating psychological factors to the risk of cancer raises public awareness to the influence of mental health in physical health outcomes, which can lead to more effective prevention strategies in public health.

### Limitations and Future Investigation

The strengths of the present review include comprehensive search performed across multiple databases, in two phases – an initial exploratory research in June 2020, refined with a second research in December 2020. The review included three psychological variables and two cancer subtypes in a single study and evaluated articles which studied the psychological factors of the participant prior to a cancer diagnosis. Furthermore, it appears to be the first systematic review to address the association between grief and cancer risk.

Some limitations can be pointed out. This systematic review does not include a meta-analysis. Only primary studies were analyzed and integrated qualitatively. In each article, the psychological variables studied were not accessed by the same measures and the associations found between the psychological factors and both breast and lung cancer were not studied cross-culturally. Other limitations include relatively short follow-up times, with most included studies having a follow-up of between 1 and 10 years – when considering that most cancers have a long latency period – and the reduced number of studies published in the last decade that met the inclusion criteria. Grief and the subsequent development of breast cancer is the least explored.

Regarding future investigation, this systematic review shows that the role of trauma in breast cancer and the role of depressive symptoms in the etiology of lung cancer need to be further explored. The associations found of trauma to breast cancer and depression to lung cancer, were in relation to different psychological outcomes, different cancer subtypes and different sexes, breast cancer more related to women and lung cancer linked to both sexes. Considering that adverse events/trauma may influence epigenetic mechanisms of disease (Ridout et al., 2018; Czamara et al., 2021; Parade et al., 2021), could this association be explained by different psychological reactions due to gender? Research is required to evaluate the relationship between specific psychological factors and the different cancer subtypes. Confounding factors such as heredity – genetic and emotional should be considered in future studies.

The recent studies using epigenetic approaches linking adverse events/trauma with mental and physical disease (Ridout et al., 2018; Lang et al., 2020; Czamara et al., 2021; Parade et al., 2021), may be the turning point to future studies shedding light on the causal mechanisms in the association of psychological factors to the risk of cancer. Likewise, in future studies, it is also important to clarify the possible mediation and moderation relationships between the psychological factors under study and the development of both breast and lung cancer.

## Conclusion

Although non-consensual, the findings from the present systematic review suggest that, in addition to the known risk factors (World Health Organization [WHO], 2020), psychological factors may play an important role in the etiology of both breast and lung cancer. The psychological factors and mind-body connection in cancer should be considered and deserves further investigation.

To consider psychological factors as a variable that affects cancer development may be fundamental to opening new avenues for prevention and intervention.

## Data Availability Statement

The original contributions presented in the study are included in the article/supplementary material, further inquiries can be directed to the corresponding author.

## Author Contributions

MP conceived and designed the study. MP and CC collected the data and all authors participated in analyzing the data. All authors read and approved the final manuscript.

## Conflict of Interest

The authors declare that the research was conducted in the absence of any commercial or financial relationships that could be construed as a potential conflict of interest.

## Publisher’s Note

All claims expressed in this article are solely those of the authors and do not necessarily represent those of their affiliated organizations, or those of the publisher, the editors and the reviewers. Any product that may be evaluated in this article, or claim that may be made by its manufacturer, is not guaranteed or endorsed by the publisher.
